# Human mitochondrial leucyl tRNA synthetase can suppress non cognate pathogenic mt-tRNA mutations

**DOI:** 10.1002/emmm.201303202

**Published:** 2014-01-10

**Authors:** Hue Tran Hornig-Do, Arianna Montanari, Agata Rozanska, Helen A Tuppen, Abdulraheem A Almalki, Dyg P Abg-Kamaludin, Laura Frontali, Silvia Francisci, Robert N Lightowlers, Zofia M Chrzanowska-Lightowlers

**Affiliations:** 1 The Wellcome Trust Centre for Mitochondrial Research, Institute for Ageing and Health, The Medical School, Newcastle UniversityNewcastle upon Tyne, UK; 2 Department of Biology and Biotechnologies “C. Darwin”, Pasteur Institute-Cenci Bolognetti Foundation, Sapienza University of RomeRome, Italy; 3 The Wellcome Trust Centre for Mitochondrial Research, Institute for Cell and Molecular Biosciences, The Medical School, Newcastle UniversityNewcastle upon Tyne, UK

**Keywords:** aminoacyl tRNA synthetase, disease, mitochondria, protein synthesis, therapy

## Abstract

Disorders of the mitochondrial genome cause a wide spectrum of disease, these present mainly as neurological and/or muscle related pathologies. Due to the intractability of the human mitochondrial genome there are currently no effective treatments for these disorders. The majority of the pathogenic mutations lie in the genes encoding mitochondrial tRNAs. Consequently, the biochemical deficiency is due to mitochondrial protein synthesis defects, which manifest as aberrant cellular respiration and ATP synthesis. It has previously been reported that overexpression of mitochondrial aminoacyl tRNA synthetases has been effective, in cell lines, at partially suppressing the defects resulting from mutations in their cognate mt-tRNAs. We now show that leucyl tRNA synthetase is able to partially rescue defects caused by mutations in non-cognate mt-tRNAs. Further, a C terminal peptide alone can enter mitochondria and interact with the same spectrum of mt-tRNAs as the entire synthetase, in intact cells. These data support the possibility that a small peptide could correct at least the biochemical defect associated with many mt-tRNA mutations, inferring a novel therapy for these disorders.

## Introduction

Mitochondria are found ubiquitously in nucleated eukaryotic cells. One of their key functions is to generate ATP via oxidative phosphorylation, in order to provide the cell with a readily usable form of energy. The machinery responsible for this process comprises five complexes, members of which are encoded by either the nuclear or the mitochondrial genome (mtDNA). Energy transduction in the cell is, thus, dependent on the precise and accurate intramitochondrial translation of the 13 mtDNA encoded polypeptides. Defects in mitochondrial metabolism are being increasingly recognized as a cause for disease. Indeed, mitochondrial disease is no longer described as a rare disorder, in part as a result of better awareness and therefore more accurate diagnosis. Studies in the north east of England indicate a clinical incidence of 9.2 in 100 000, with a further 16.5 in 100 000 at risk of developing mtDNA disease before retirement (Schaefer *et al*, 2008). Pathogenesis can arise from defects either in nuclear encoded proteins that function in the mitochondria (Smits *et al*, 2010; Chrzanowska-Lightowlers *et al*, 2011; Rotig, 2011; Keogh & Chinnery, 2013) or from mutations in the mtDNA itself (Chinnery & Turnbull, 2000; Greaves *et al*, 2012). The ease of sequencing the comparatively small mitochondrial genome (Anderson *et al*, 1981) has allowed a comprehensive characterization of pathogenic mtDNA mutations, establishing that the majority of these mutations reside in the mitochondrial tRNA genes. These would be predicted to impair mitochondrial protein synthesis and since it is currently not possible to manipulate human mtDNA, researchers have explored other ways to overcome defects of mt-tRNAs. Such approaches have often started with yeast as a model system, in which it has been possible to isolate nuclear suppressor factors that are able to rescue the defective phenotype of mt-tRNA mutants (Rinaldi *et al*, 1997; Feuermann *et al*, 2003; De Luca *et al*, 2006). In addition, in a previous study with human cells lines harbouring a pathogenic mt-tRNA^val^ mutation (m.1624C>T) we showed that overexpression of the cognate aminoacyl tRNA synthetase, VARS2, could partially suppress the consequent molecular defect (Rorbach *et al*, 2008). This work demonstrated that the mutation caused destabilisation of the uncharged mt-tRNA^val^, such that the transmitochondrial cybrid line carrying the mutation had severely depleted steady state levels of mt-tRNA^val^. Following induction, the higher levels of VARS2 increased the proportion of charged mt-tRNA^val^, thereby increasing the steady state levels (Rorbach *et al*, 2008). Other examples of suppression via overexpression of a cognate tRNA synthetase in human cell lines now exist. One of these is the leucyl tRNA synthetase (Herbert *et al*, 1988), overexpression of which has been reported to suppress the effect of an m.3243A>G mutation in human cells (Li & Guan, 2010). In contrast to the m.1624C>T mutation that is associated with Leigh disease (McFarland *et al*, 2002), this mutation causes a different mtDNA disease, MELAS (Shaag *et al*, 1997).

Genetically correcting an mtDNA defect is not currently a therapeutic option. Moreover human mtDNA encodes 22 different tRNAs, therefore, it would be ideal if a single approach could be developed that would be equally effective with any pathogenic mt-tRNA mutation. With relevance to this, we were aware of an earlier published observation that the yeast mt-leucyl tRNA synthetase (NAM2) possessed generic RNA binding capability (Labouesse *et al*, 1987; Herbert *et al*, 1988) and subsequently that it could suppress non-cognate mt-tRNA mutations in yeast (Montanari *et al*, 2010). We predicted that the human orthologue, LARS2, might also have the potential to counteract generic mt-tRNA defects in human cells. Here we illustrate that in human cells, overexpression of the non-cognate mitochondrial leucyl tRNA synthetase can indeed have a similar suppressive effect, ameliorating the biochemical dysfunction. This observation has an important clinical relevance as more than 200 pathogenic mt-tRNA mutations have been reported, at least 116 since 2003 (Yarham *et al*, 2011) and due to the intractability of the human mitochondrial genome there is currently no genetic therapy available. The data and approach presented here may provide a way forward towards a single therapy that could ameliorate the clinical condition arising from all mt-tRNA mutations.

## Results

**Figure 1:**
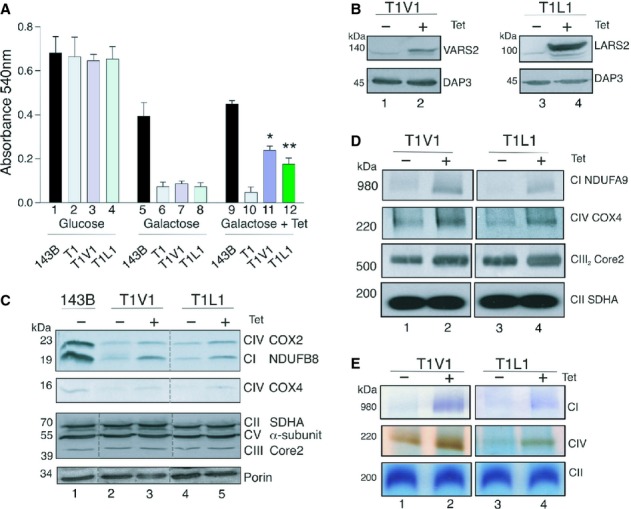
In each case aaRS overexpression was induced by 3 days tetracycline (Tet) treatment, indicated by + or − symbols. All data and images represent a minimum of 3 independent experiments. Cell growth under glycolytic or respiratory conditions with or without overexpression of aaRS. Equal numbers of 143B.206 Rho^+^, T1, T1V1 and T1L1 cells were seeded in medium containing either glucose or galactose as the sole sugar source. The extent of growth and viability of each cell line with or without aaRS overexpression was determined at 72 h using the neutral red assay. Statistically significant comparisons for uninduced and induced cells are indicated with * ( *P* = 0.016), ** ( *P* = 0.0086) *n* = 3.Overexpression of aaRS. Mitochondrial proteins were isolated from uninduced and aaRS overexpressors. Mitochondrial lysates from T1V1 (100 μg) and T1L1 (50 μg) were subjected to western blot analysis using antibodies against VARS2L or FLAG to confirm overexpression. Mitochondrial ribosomal protein DAP3 was used to confirm equal loading.Analysis of steady state levels of OXPHOS proteins following aaRS induction. Cell lysates (15 μg) from 143B.206 Rho^+^ and T1V1 and T1L1 cells with or without induction were subjected to western blot analysis. Membranes were probed with antibodies directed against OXPHOS proteins and porin as a loading control.Blue Native-PAGE analysis of respiratory chain complex levels. Mitochondria (25 μg) from uninduced and induced T1V1 and T1L1 cells were solubilised for BN-PAGE. Subsequent western blot analysis used antibodies against complex I (NDUFA9), complex IV (COX4), complex III (CORE2) and as a non-mitochondrially encoded control, complex II (SDHA).In gel enzyme activity assay of respiratory chain complexes. BN-PAGE was performed on solubilised mitochondria (50 μg) from uninduced and induced T1V1 and T1L1 cells. Enzyme activities for complexes I, IV and II were examined.

As described above, we previously showed that overexpression of VARS2 could partially restore the steady state levels of mt-tRNA^val^ resulting from the m.1624C>T mutation (Rorbach *et al*, 2008). However, this original cybrid population, generated from a fusion of 143B.206 ρ^0^ cells with patient cytoplasts homoplasmic for the m.1624C>T Leigh disease mutation, did not recapitulate the profound biochemical phenotype found in the patient's skeletal muscle (Rorbach *et al*, 2008). We therefore aimed to exploit the aneuploid nature of the 143B.206 ρ^0^ parental cell line and identify whether isolating clones from the population would expose a stronger metabolic defect in a subset of cells. Clones were derived by serial dilution in glucose. Following expansion, the clones were then independently assessed for growth in galactose, a carbon source that forces cells to use oxidative phosphorylation. Of the 67 clones that were tested only six demonstrated impaired respiratory capacity when challenged to grow on this carbon source. Although a number of these clones were selected for transfection with constructs that would allow inducible expression of aminoacyl tRNA synthetases (aaRS), the data presented here are the characterization of a representative clone (T1) (Fig 1A lanes 2, 6 and 10). Inducibility of aaRS expression was a key factor in the experimental design, as due to the aneuploid nature of 143B.206 cells, it was critical that the behaviour of exactly the same clone could be examined with and without aaRS overexpression. In order to confirm that the respiratory deficiency was attributable to the mt-tRNA^val^ mutation, we transfected the respiratory deficient T1 line with VARS2 (designating it T1V1) and monitored growth on galactose. On induction of VARS2 expression, we observed suppression of the m.1624C>T defect as determined by increased growth on galactose (Fig 1A lanes 7 and 11).

### Overexpression of LARS2 can partially rescue the growth phenotype and molecular defects associated with a non-cognate mitochondrial tRNA

The equivalent m.1624C>T *MT-TV* mutation, as well as the MELAS m.3243A>G *MT-TL1* mutation, had previously been introduced into yeast mtDNA and the mitochondrial leucyl or valyl tRNA synthetase were each independently overexpressed. Interestingly, suppression was achieved for the *MT-TV* mutation using the non-cognate leucyl tRNA synthetase (Montanari *et al*, 2010). More recently it has been shown, again in yeast (Francisci *et al*, 2011), that not only do non-cognate aaRS have the ability to suppress the defects caused by mt-tRNA mutations, but that isolated domains of the aaRS alone could also have a similar effect (Montanari *et al*, 2010; Francisci *et al*, 2011). Since these investigations had been performed in yeast, we decided to use the T1 line harbouring the mt-tRNA^val^ mutation to see if similar suppression by a non-cognate aaRS could be achieved in human cells. In a parallel fashion to the yeast work, we transfected T1 with a FLAG tagged version of the human LARS2, (T1L1). This line together with the T1V1, T1 and 143B.206 ρ^+^ parental cells were routinely propagated on glucose and displayed similar doubling times (Fig 1A lanes 1–4). When transferred to galactose the parental 143B.206 ρ^+^ line continued to grow but with an extended doubling time. In contrast the T1 and aaRS-transfected derivatives were all either unable to grow or showed negligible growth unless cells were induced to express aaRS (Fig 1A *cf* lanes 7 and 11; 8 and 12). In each cell line the induction of the relevant aaRS was confirmed by western blot analysis (Fig 1B *cf* lanes 1 and 2; 3 and 4).

**Figure 2:**
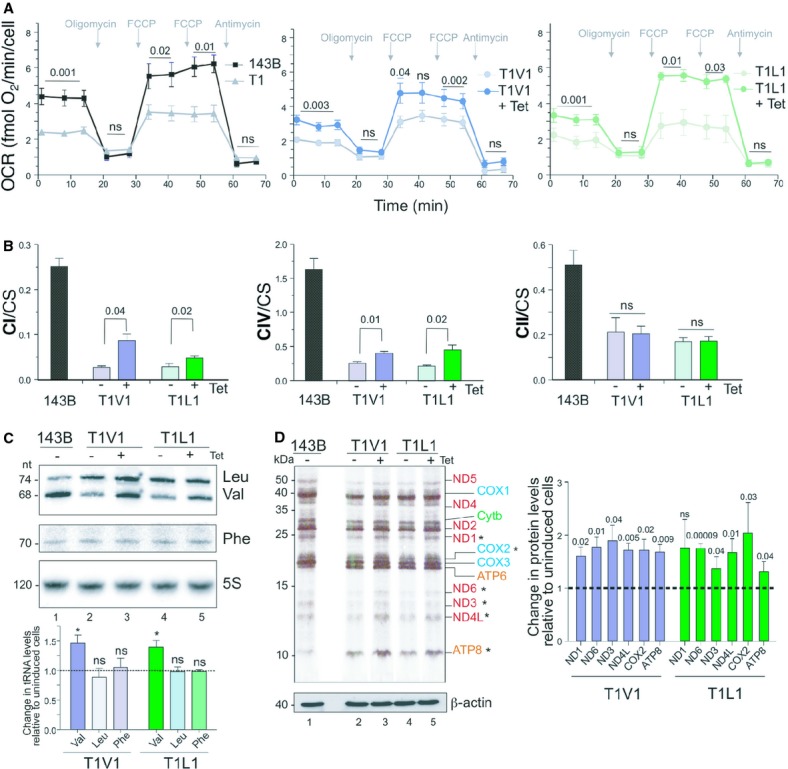
In each case aaRS overexpression was induced by 3 days tetracycline (Tet) treatment (indicated by + or −), except for *XF24 analysis*. All data and images represent a minimum of 3 independent experiments. Statistically significant *P* values are as indicated. Oxygen consumption by XF24 analyzer. Representative traces of oxygen consumption rates performed under basal conditions, following the addition of oligomycin (1 μg/ml), uncoupler FCCP (1.5 μM then 3 μM) and antimycin A (2.5 μM) are presented for 143B.206 Rho^+^, T1, T1V1 and T1L1 cell lines with or without 2 days aaRS overexpression.Respiratory chain activity improves on cognate aaRS or LARS2 overexpression. Mitochondria were isolated from 143B.206 Rho^+^ cells and T1V1 and T1L1 transfectants with and without aaRS induction. Enzyme activities of complexes I, IV and II were measured spectrophotometrically and are presented as ratios against citrate synthase.Steady state levels of mt-tRNA^val^ increase following VARS2 or LARS2 overexpression. Equal amounts of RNA (4 μg) isolated from 143B.206 Rho^+^ cells and T1V1 and T1L1 transfectants with and without aaRS induction were analysed by high resolution northern blotting. Probes were specific for mitochondrial tRNA^leu(UUR)^, tRNA^val^, and tRNA^phe^ or 5S RNA as a loading control. Densitometric measurements of mt-tRNA^leu(UUR)^, tRNA^val^, and tRNA^phe^ in uninduced and induced T1V1 and T1L1 transfectants were normalised to the 5S RNA. Data are expressed as percentages of mt-tRNA in induced cells over mean uninduced cell values and presented below * *P* < 0.02.Synthesis of mitochondrially encoded proteins increases upon aaRS overexpression. Mitochondrially encoded proteins in 143B.206 Rho^+^, T1V1 and T1L1 cells with and without aaRS overexpression, were pulse labeled with ^35^S-methionine and separated by 15% SDS–PAGE. Below the autoradiogram is a western blot of the same membrane probed for β-actin as loading control. Increases in protein synthesis post induction are indicated (right panel) by densitometric quantification of individual RC subunits (designated by *), relative to-Tet and normalized to β-actin.

Next we measured the steady state levels of respiratory complex proteins to determine whether the partial growth defect suppression truly reflected an improvement in respiratory competence. Following aaRS induction, the levels of mitochondrially encoded COX2 increased. An increase was also observed in the levels of NDUFB8, a sensitive marker of Complex I (CI) assembly. Nuclear encoded COX4 also appeared to have a modest increase in steady state levels (Fig 1C *cf* lanes 2 and 3; 4 and 5). Complex II is encoded entirely by the nuclear genome and showed no change when probed for SDHA (Fig 1C). Since the steady state levels are not always a true indicator of complex assembly, Blue Native PAGE was performed with either subsequent western analysis or in gel activity assays. Here again CII appeared unchanged in T1V1 or T1L1 induced cells, however an increase in assembled CI and CIV could be seen, with a more modest increase in CIII (Fig 1D *cf* lanes 1 and 2; 3 and 4). These increases in complex formation were reflected in the in gel activities for the cell lines expressing either VARS2 or LARS2 (Fig 1E *cf* lanes 1 and 2; 3 and 4). To directly measure the oxygen consumption we used microscale oxygraphy. Overexpression of either VARS2 or LARS2 resulted in partial recovery of basal and maximal respiration rates, while respiration rates after oligomycin and antimycin inhibition were not altered (Fig 2A). To more accurately assess the recovery in respiratory chain complex activities, mitochondria were isolated from uninduced and aaRS overexpressing cells, on which spectrophotometric assays were performed for complexes I, II and IV. There was no change in CII activity, whilst induction of aaRS significantly increased CI and CIV activity, where in most cases there was at least a doubling of activity (Fig 2B).

The analyses thus far were directed at recovery of function rather than any direct consequence on the mutant tRNA^val^. Steady state levels of mt-tRNAs were therefore assessed by high resolution northern. The level of mt-tRNA^val^ was determined and compared to those of mt-tRNA^phe^ and mtRNA^leu(UUR)^. Over-expression of either VARS2 or LARS2 had no effect on either of the two wild type tRNAs but did increase the level of the mutated tRNA^val^ transcript (Fig 2C *cf* lanes 2 and 3; 4 and 5). Densitometric analysis indicated that in each case, overexpression of aaRS increased the mt-tRNA^val^ to approximately 150% of the levels in uninduced cells (Fig 2C lower panel).

The impact of aaRS overexpression on *de novo* synthesis of mitochondrial protein was also assessed. Metabolic labeling was performed on induced and uninduced T1V1 and T1L1 alongside 143B.206 ρ^+^ parental cells. Following induction of either aaRS, there was an overall increase in labeled protein of approximately 1.5 fold, which corresponded to the increase seen in steady state levels of the mutated mt-tRNA^val^ under the same conditions. Densitometric analysis of individual products indicated 1.6–1.9 fold increases after VARS2 induction (Fig 2D *cf* lanes 2 and 3) and 1.3–2 fold increases after LARS2 overexpression (Fig 2D *cf* lanes 4 and 5).

### LARS2 C-term 67 residues can bind to mt-tRNA in intact cells

**Figure 3:**
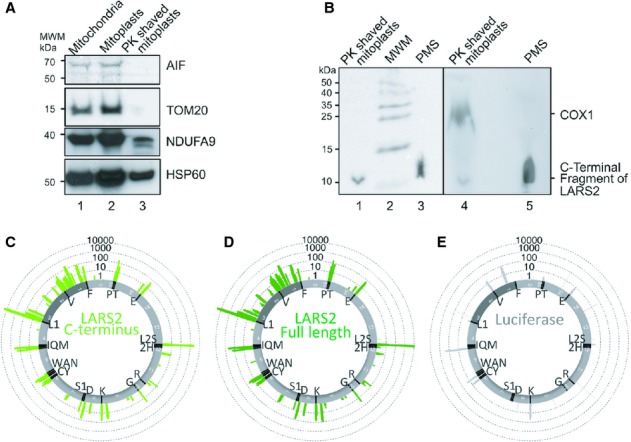
In each case overexpression of the LARS2 FLAG tagged C-terminus was induced by 3 days tetracycline (Tet) treatment. Samples presented in panels A and B were separated on a single 16% Tricine PAG. Post transfer the membrane was cut to allow probing with different antibodies, positions of the molecular weight markers (MWM) are indicated. Samples in C, D and E represent RNA binding determined by crosslinking/immunoprecipitation (CLIP). Derived data is presented as IonTorrent reads aligned against the mtRNA genes, which are depicted as in circular mtDNA. Concentric circles indicate a log scale of number of reads / site. AProteinase K (PK) shaving of mitoplasts confirms loss of OMM and IMS but retention of IMM and matrix fractions. Mitochondria (50μg, lane 1), mitoplast (75μg, lane 2) and shaved mitoplast (50μg, lane 3) fractions were analysed by western with the antibodies indicated.BLARS2 C-terminal peptide is localized to the inner mitochondrial compartment. The shaved mitoplasts (˜1mg of preparation presented in panel A; lanes 1 and 4) and TCA precipitated postmitochondrial supernatant (PMS; lanes 3 and 5) were probed for the presence of the LARS2 C-terminus. COXI was used as a marker to confirm presence of inner mitochondrial proteins that were absent from the PMS fraction. The poor resolution is due to the large amount of protein loaded in this well. Left Panel directly visualized by BioRad Chemidoc MP, right panel visualized by autoradiography.C–ECLIP derived mt-RNA binding capacity. (C) Physiological RNA binding by the LARS2 C-terminal peptide; (D) Physiological RNA binding by the full length LARS2; (E) As a control, RNA binding by the mitochondrially targeted FLAG tagged luciferase was also determined.

As mentioned previously, there is evidence that a C terminal fragment alone, of the mitochondrial leucyl tRNA-synthetases from either yeast or human, is sufficient to suppress the respiratory defects caused by mutations in yeast mt-tRNAs Leu, Val or Ile (Francisci *et al*, 2011). Structural studies indicate that this C terminal region of the tRNA synthetase folds into a compact domain that is flexibly linked to the rest of the structure. It also exhibits tRNA binding activity, recognising the mt-tRNA elbow shape rather than sequence (Tukalo *et al*, 2005). We therefore determined whether this fragment alone was able to bind mt-tRNAs other than mt-tRNAleu(UUR) in human cells. To be sure that we were detecting physiological binding we used the CLIP method. Intact cells are exposed to UV to preserve RNA/protein interactions prior to mitochondrial isolation and immunoprecipitation of the protein of interest. Cells were therefore transfected with various LARS2 constructs that were FLAG tagged to facilitate immunoprecipitation. Although the construct encoding the C-terminal 67 amino acids of LARS2 was FLAG tagged it had no additional N-terminal sequence to promote mitochondrial targeting. However, analysis of the amino acid sequence of the C-terminal peptide using predictive algorithms (including http://www.tcdb.org/progs/helical_wheel.php) does indicate a helix with a clear hydrophobic face but this is only weakly amphipathic. Western blotting of this small fragment confirmed weak but consistent localization to mitochondria after induction. This was performed initially on eluates of FLAG mediated immunoprecipitation from isolated organelles (supplementary Fig S1). We then repeated these studies to identify the exact submitochondrial compartment the C term fragment was able to access. With this aim, mitochondria from cells expressing the C-terminal fragment (Fig 3A lane 1) and mitoplasts were prepared (Fig 3A lane 2) as described in Materials and Methods. These were proteinase K shaved to confirm loss of outer membrane (TOM20), and intermembrane space (AIF) proteins, with retention of the inner membrane (NDUFA9) and matrix (HSP60) proteins (Fig 3A lane 3; 3B lanes 1 and 4). These shaved mitoplasts (Fig 3B lanes 1 and 4) and the postmitochondrial supernatant (PMS; Fig 3B. lanes 3 and 5) were western blotted to confirm that the C-terminal fragment does enter mitochondria. This intramitochondrial matrix localization was further confirmed by the CrossLinking-ImmunoPrecipitation data. CLIP analysis of mtDNA-encoded cDNA sequences rescued following crosslinking to physiological RNA substrates and subsequent immunoprecipitation indicated that although the C-terminus showed a significant preference for the two mt-tRNA^leu^ species, it was also able to bind a number of other mt-tRNA species (Fig 3C). Further analysis showed that these binding events were virtually superimposable on the binding pattern of the full length LARS2 (Fig 3D), consistent with suppressive effect seen here and previously in yeast (Francisci *et al*, 2011). FLAG tagged luciferase was used as a control and showed negligible binding to any mt-RNA (Fig 3E).

### Not all mitochondrial tRNA synthetases can rescue the m.1624C>T mutation

**Figure 4:**
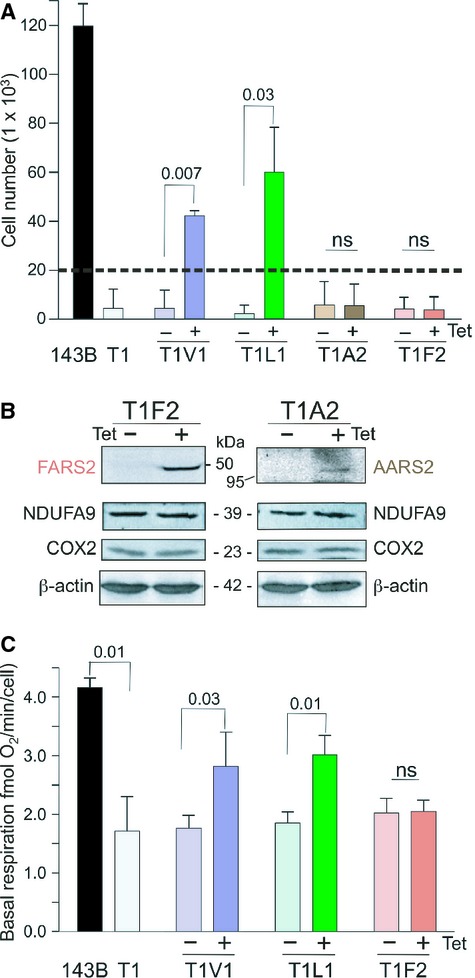
In each case aaRS overexpression was induced by tetracycline (Tet) treatment, indicated by + or − symbols. All data and images represent a minimum of 3 independent experiments. Statistically significant *P* values are as indicated.
Growth rates for cell lines with or without aaRS overexpression. 143B.206 Rho^+^, T1 cells and T1 transfectants for VARS2 (T1V1), LARS2 (T1L1), AARS2 (T1A2) and FARS2 (T1F2) were seeded at 2 × 10^4^ (indicated by dashed line) in medium containing galactose. Cells were counted at 72 h with (+) or without (−) aaRS overexpression. Data are represented as mean ± s.d.Overexpression of neither alanyl-nor phenylalanyl tRNA synthetase changes steady state levels of respiratory protein. Western blots of cell lysates (25 μg) from T1A2 and T1F2 cells with or without 3 days aaRS overexpression were probed with antibodies directed against FLAG tag, OXPHOS proteins and β-actin as a loading control. Lengthy exposures were required to ensure detection of COX2 signals.Overexpression of neither alanyl-nor phenylalanyl tRNA synthetase rescues respiratory defect. Basal respiration rates (mean ± s.d.) were determined for 143B.206 Rho^+^, T1 cells and T1V1, T1L1 and T1F2 transfectants with or without aaRS 2 days overexpression.

Finally, to determine whether suppression of the *MT-TV* mutation could be elicited by overexpression of other mitochondrial aaRS, we transfected T1 with constructs allowing inducible expression of two mitochondrial class II synthetases, either alanyl (AARS2; T1A2) or phenylalanyl (FARS2; T1F2) tRNA synthetase. Overexpression of AARS2 or FARS2 protein had no effect on galactose growth (Fig 4A). Analysis of the steady state level of the mtDNA encoded COXII was performed and confirmed that there was no change following overexpression of either of these aaRS proteins (Fig 4B). This lack of suppression was further confirmed by microscale oxygraphy measurements, where overexpression of non-cognate aaRS, other than LARS2, could not rescue the biochemical deficiency of the T1 mt-tRNA^val^ mutation (Fig 4C).

## Discussion

The data presented here indicate that human mt-leucyl tRNA synthetase (LARS2) has the ability to suppress the biochemical defects seen in mitochondrial metabolism that can result from an mt-tRNAval mutation. It has been previously documented that in cell lines harbouring mitochondrial tRNA mutations, partial or full suppression can be achieved by overexpression of the cognate aminoacyl tRNA synthetase (Park *et al*, 2008; Rorbach *et al*, 2008; Li & Guan, 2010). Moreover, there is a naturally occurring example of suppression of a homoplasmic m.4277T>C *MT-TI* mutation by overexpression of the cognate tRNA synthetase. Here, a family carrying this homoplasmic mutation showed varied levels of penetrance of the clinical and biochemical defect. Tissue specific investigations of the index case and the clinically unaffected mother demonstrated that the mother had naturally elevated levels of isoleucyl-tRNA synthetase (IARS2) that suppressed the phenotype resulting from the otherwise pathogenic mutation (Perli *et al*, 2012). Why then should LARS2 have the ability to suppress mutations in non-cognate tRNAs? It appears that LARS2 may have developed a bifunctional role in the cell. Aminoacyl tRNA synthetases are evolutionarily critical proteins that were probably involved in the transition from the RNA world, and appear to have recruited new cell functions (Rho *et al*, 2002; Sarkar *et al*, 2012; Yao *et al*, 2013). Indeed, the yeast LARS2 equivalent, LeuRS was not originally identified by its ability to aminoacylate its cognate tRNA, consistent with it having developed multiple functions. It was identified through a screen originally devised to isolate proteins that when mutated, could compensate for mitochondrial splicing defects in yeast (Dujardin *et al*, 1980). This screen revealed a number of proteins that showed ‘nuclear accommodation of mitochondria’ and their genes were accordingly classified as NAM1, NAM2 and so forth (Dujardin *et al*, 1980). It was only following subsequent characterisation that NAM2 was confirmed as encoding the mitochondrial leucyl-tRNA synthetase (LeuRS) (Herbert *et al*, 1988). Further inspection of the protein domains was strongly suggestive of an RNA binding activity (Labouesse *et al*, 1985, 1987) that would be consistent with a role in splicing.

The importance of this observation to the work presented here is that splicing factors often act by stabilizing the structure of the catalytically active core of self-splicing RNA transcripts. Importantly, the group I intronic sequences that LeuRS recognizes and is directly involved in splicing in yeast, are highly structured (Michel *et al*, 1982; Labouesse, 1990) and are distinct from the structures of group II introns (Bonen & Vogel, 2001). It is therefore possible that LeuRS may recognize common structures in mt-tRNAs that are similar to those conserved in group I introns (Michel *et al*, 1982; Labouesse, 1990). Such an hypothesis has been previously proposed by Myers *et al* (Myers *et al*, 2002) to explain the recognition of group I introns by another type 1 mitochondrial aminoacyl tRNA synthetase, tyrosyl-tRNA synthetase (Akins & Lambowitz, 1987; Majumder *et al*, 1989; Kittle *et al*, 1991). Clearly, additional sequence or structural specificity will be required for splicing, but at least for LeuRS the result may be a generic ability to bind to non-cognate mt-tRNAs.

The splicing function and thus part of the RNA binding activity was initially identified in the yeast LeuRS by deletion analysis, showing that this activity resided in the C terminal region of the protein, and that these changes essentially had no impact on the aminoacylation activity (Li *et al*, 1996). This C-terminal domain is highly conserved, in amino acid sequence and in structure, from Archaea to human mitochondria, but has been lost in the mammalian cytosolic forms (Cusack *et al*, 2000; Tukalo *et al*, 2005; Hsu *et al*, 2006). Further, although there is no requirement for intron splicing in human mtDNA expression, the human LARS2 is still able to restore splicing in a yeast NAM2 delete strain (Houman *et al*, 2000).

More recent work has highlighted how this C-terminal is working. Although the ˜60 amino acids in this C-terminal extension form a unique domain that appears to have evolved to optimize its multiple roles in aminoacylation and splicing (Hsu *et al*, 2006), the C-terminal domain of LeuRS does not interact with the anticodon of its cognate tRNA (Larkin *et al*, 2002). This region interacts directly with the elbow of the L-shaped tRNA and is based more on structural recognition of the tRNA shape rather than through specific base-base interactions (Tukalo *et al*, 2005; Hsu *et al*, 2006).

Why should an aaRS, which should have tRNA sequence specificity, develop recognition of shape over sequence? The answer may be that despite their predicted stable ‘cloverleaf’ structure, organellar tRNAs appear to have greater structural relaxation than bacterial tRNAs (Fender *et al*, 2012) so there is arguably a greater need for interacting proteins to stabilize their shape. In support of this is the observation that a number of *in vitro* aminoacylation assays show greater activity if a polyamine is preincubated together with the substrate tRNA (Bullard *et al*, 1999). In particular, the addition of spermine is believed to stabilize the cloverleaf structure of certain tRNAs (Pegg, 1988). Curiously this enhancement of aminoacylation was not observed in assays with the human mt-leucyl tRNA synthetase (Bullard *et al*, 2000). This would support our hypothesis that LARS2 itself has a stabilising effect on the tRNA substrate, which in turn will promote more efficient aminoacylation, particularly in the case of destabilizing mutations.

Finally, is this non-cognate mt-tRNA stabilizing function unique to the C-terminal of LARS2 ? The data in this paper only minimally address this issue. We find that representatives of the class II mitochondrial aaRS are unable to even partially rescue growth of the *MT-TV* mutation. However, the accompanying paper shows that at least one other type I tRNA synthetase, VARS2 is capable of cross rescuing non-cognate mt-tRNA defects in human cell lines. This may also be the case for other mitochondrial aaRS, but we are unable to comment directly on this. However, it is interesting to consider that leucyl tRNA synthetase belongs to a subfamily of large monomeric synthetases that includes valyl-, and isoleucyl-tRNA synthetases. Further, the yeast mitochondrial orthologues of each of these proteins has been shown to suppress non-cognate mt-tRNA mutations (Francisci *et al*, 2011). Perhaps this subfamily of aminoacyl tRNA synthetases has retained an RNA binding feature that can be exploited therapeutically. Preliminary analysis of hydropathy profiles or primary amino acids sequences showed no obvious motifs but more detailed investigation of whether there are any characteristics shared by members of this family will form part of our ongoing studies.

In summary, we have shown that overexpression of the human mitochondrial leucyl tRNA synthetase can be used to suppress the defects caused by a destabilizing mutation in mt-tRNA^val^, a non-cognate mt-tRNA. Further, CLIP data of RNA binding derived for the C-terminus of LARS2 demonstrated an almost identical binding spectrum to mitochondrial tRNAs as the full length LARS2. We believe that this provides good evidence for the potential therapeutic use of the C-terminus of LARS2 for most pathogenic human mt-tRNA mutations.

## Materials and Methods

### Cell culture

Human 143B.206 Rho^+^, cybrid derivatives and Flp-InT-Rex-293 cells (HEK293T; Invitrogen Life Technologies Ltd, Paisley, UK) cells were cultured (37°C, humidified 5% CO_2_) in DMEM (Sigma-Aldrich Company Ltd., Dorset, UK) supplemented with 10% (v/v) foetal calf serum (FCS), 1× non-essential amino acids (NEAA) and 2 mM L-glutamine and where appropriate with the addition of 10 μg/ml Blasticidin^S^ and Hygromycin^B^ (100 μg/ml) post transfection to select for successful integration. For growth on respiratory substrates, the medium contained glucose-free DMEM (Gibco Life Technologies Ltd, Paisley, UK), 0.9 mg/ml galactose, 1 mM sodium pyruvate, 10% (v/v) FCS, NEAA and 2 mM L-glutamine and 50 μg/ml uridine. In each case induction of aaRS in each cell line was achieved by addition of 1 μg/ml tetracycline for 3 days.

### Neutral red cell counting assay

The number of living cells was evaluated by automated counting and neutral red assay as described in Repetto *et al* (2008).

### Cell lysate, westerns and antibodies

SDS–PAGE analysis was performed on cell lysate extracted from cultured cells as described previously (Soleimanpour-Lichaei *et al*, 2007). After electrophoresis, gels were transferred to a PVDF membrane (GE Healthcare, Amersham, UK) and processed for immunoblotting. The following commercially available antibodies were used: NDUFB8, NDUFA9, Core2, COXI, COXII, COXIV, SDHA, Complex Vα subunit, porin (MitoSciences, Eugene, OR, USA); cytochrome *c* (BD Biosciences, Oxford, UK); β actin, FLAG (Sigma Life Science); DAP3 (Abcam, Cambridge, UK); TOM20 (Santa Cruz, Heidelberg, Germany); AIF (NEB, Hitchin, UK); HSP60 (BD Biosciences); VARS2 custom synthesized; all secondaries were HRP conjugated (Dako, Stockport, UK).

### Spectrophotometric activity assays

The activities of complex I, II and CIV and citrate synthase, as a mitochondrial matrix marker, were determined in isolated mitochondria as previously described (Kirby *et al*, 2007).

### Oxygen consumption measurements

Measurement of intact cellular respiration was performed using the XF24 analyzer (Seahorse Bioscience, Saint Marcell, France). Cells were plated at a density of 10 000 cells/well on XF24 tissue culture plate. After 4 h cells were induced by adding tetracycline (1 μg/ml), which was retained in the culture media for 2 days. Prior to the respiration assay, cells were rinsed and cultured in assay medium supplemented with 5 mM glucose 10 mM sodium pyruvate (Sigma) according to manufacturer's protocol. Cells were incubated at 37°C in a CO_2_ free incubator for 1 h prior to measurement. Oxygen consumption was measured under basal conditions, and in the presence of oligomycin, a complex V inhibitor (1 μg/ml), the complex III inhibitor antimycin (2.5 μM), and mitochondrial uncoupler FCCP (first addition at 1.5 μM, second addition at 3 μM, Sigma) to assess maximal oxidative capacity. To normalize respiration rates to cell number, cells were fixed with 4% paraformaldehyde and counted after nuclear staining with Hoechst 33258 (1 μg/ml, Invitrogen).

### Mitochondrial preparation

Mitochondria were isolated using superparamagnetic microbeads as described previously (Hornig-Do *et al*, 2009). For the confirmation of intramitochondrial localization of the C-terminal fragment of LARS2, preparations were as follows. HEK-293T cells were homogenized (glass:Teflon dounce homogeniser, 20 passes) on ice in isolation buffer (10 mM Tris–HCl, pH 7.4, 0.6 M mannitol, 1 mM EGTA and 0.1% BSA). Aggregates were removed by centrifugation at 400 g for 10 min at 4°C. Mitochondria were pelleted at 11 000 g for 10 min at 4°C. The post-mitochondrial supernatant was TCA precipitated before resuspension in dissociation buffer. Pelleted mitochondria were washed (in isolation buffer) and mitoplasts generated by hypotonic shock in ice cold 5 mM Tris–HCl pH 7.4, 1 mM EDTA. Mitoplasts were Proteinase K treated (5 μg/mg of mitochondria) in isolation buffer lacking BSA, for 30 min at 4°C. Reactions were then stopped by the addition by 1 mM PMSF and washed in isolation buffer lacking BSA.

### Metabolic labelling of mitochondrial translation products

Essentially as described in Chomyn (1996). Cells were labeled with ^35^S-methionine (0.05 mCi/25 cm^2^ flask) for 1.5 h in methionine-and cysteine-free DMEM supplemented with 100 μg/ml emetine and 5% dialyzed FBS (Chomyn, 1996). Proteins (40 μg) were electrophoresed through a 15% denaturing gel. Proteins were transferred to PVDF membranes before autoradiography where band intensities corresponding to mitochondrial translation products were quantified densitometrically (ImageQuant/Molecular Dynamics, GE Healthcare, Amersham, UK), then probed with β actin to ensure equal loading of cell lysates.

### BN-PAGE and enzymatic in-gel-activity

BN-PAGE analysis was performed as previously described (Hornig-Do *et al*, 2012). Briefly, mitochondria were solubilised by dodecylmaltoside (DDM, Sigma) at 2 g/g protein and incubated for 20 min on ice. The supernatant was collected after 20 min centrifugation at 25 000 *g*. To resolve individual complexes and smaller supercomplexes, 25 μg of DDM treated mitochondrial membrane proteins were separated on 4.5–16% gels (Wittig *et al*, 2006). Post electrophoresis, complexes were transferred to PVDF membranes and sequentially probed with indicated antibodies. In-gel complex activity assays were carried out to estimate the activity of CI, CII and CIV as described (Calvaruso *et al*, 2008).

### High resolution northern

Total RNA was extracted using TRIzoLTM (Invitrogen). RNA (4 μg) was electrophoresed through 15% denaturing polyacrylamide gel, electroblotted onto GeneScreen Plus membrane (Perkin Elmer, Cambridge, UK) and fixed by UV crosslinking prior to hybridization with radiolabelled probes. Probe fragments were generated by PCR using the following primer pairs. Human mt-tRNA^leu[UUR]^ forward 5′-TATACCCACACCCACCCAAG-3′, and reverse 5′-GCGATTAGAATGGGTACAAT-3′; human mt-tRNA^phe^ forward 5′-CCAAACCCCAAAGACACCC-3′ and reverse 5′-GAACGGGGATGCTTGCATG-3′; human mt-tRNA^val^ forward 5′-CTGGAAAGTGCACTTGGACG-3′ and reverse 5′-GGGTAAATGGTTTGGCTAAGG-3′; human 5S forward 5′-GTCTACGGCCATACCACCCTG-3′ and reverse 5′-AAAGCCTACAGCACCCGGTAT-3′. Purified amplicons were radiolabelled with [α-^32^P] dCTP (3000 Ci/mmol, GE Healthcare) using random primers. Unincorporated nucleotides were removed by Sephadex G-50 column (GE Healthcare). Hybridisation was carried out as previously described (Rorbach *et al*, 2008).

### Statistics

Results are given as means ± standard deviation and were compared using unpaired *T*-tests assuming unequal distribution. A significance level of *P* < 0.05 was considered to be statistically significant *; *P* < 0.01 = **; *P* < 0.001 = ***.

### AARS expression constructs and cybrid transfection

To generate inducible expression variants of the T1 line carrying the mt-tRNA^val^ mutation, each transfection was a combination of pcDNA6/TR and pcDNA5/FRT/TO (both Invitrogen) containing the gene for VARS2 (Q5ST30-1 OMIM:612802, Kazusa clone KIAA1885), LARS2 (NP_056155.1 OMIM:604544), FARS2 (NP_006558.1 OMIM: 611592, IMAGE clone 5088776/MGC 31883) or AARS2 (NP_065796.1 OMIM: 612035, Kasuza clone KIAA1270). VARS2 construct was as defined in (Rorbach *et al*, 2008). All other aaRS had inframe C terminal FLAG tag encoded and were generated by PCR with primers containing restriction sites (underlined) to allow ligation into pcDNA5/FRT/TO (Invitrogen). In each case the complete open reading frame was amplified. The entire LARS2 ORF was amplified from IMAGE clone 4822546 using For 5′-CACACAGGATCCCCTTCTCACCTTCTGAAG-3′ / Rev 5′-ACTCGACTCGAGCTACTTATCGTCGTCATCCTTGTAATCATCTTGCACCAGGAAGTTG-3′; FARS2, IMAGE clone 5088776/MGC 31883 using For 5′-CACACAGGATCCGCAGAGTGTGCCACCCAAAG-3′ / Rev 5′-CACACAGGATCCAGCCTGAGTGAAGTGGTGAC 3′; AARS2, Kasuza clone KIAA1270 using For 5′-CACACAGATATCGATGGCAGCGTCAGTG-3′ / Rev 5′-CACACAGCGGCCGCCTACTTATCGTCGTCATCCTTGTAATCGAGCTGGCTGAGGGCATAGG. The C-terminal domain (encoding the final 67 amino acids) of *LARS2* gene was amplified from the full length *LARS2* of pcDNA5/FRT/TO LARS2 plasmid using the forward primer For 5′-CGGGATCCCG**ATG**GAGGTTGTCCAGATGGCAGTTC-3′, which contained the additional ATG codon (in bold) and a BamH1 (underlined) restriction site. The reverse primer Rev 5′-CGGGATCCCGTCA**GATATC**TTGCACCAGGAAGTTG-3′, also contained BamH1 and EcoRV restriction sites (underlined/bold). The PCR product was BamHI restricted and cloned into BamHI restriction site of the pcDNA5/FRT/TO plasmid generating an intermediate step vector ‘pcDNA5-CTerm’. Independently we designed two complementary oligomers (For 5′-GATATCGATTACAAGGATGACGACGATAAG**TAG**GATATC-3′/Rev 5′-GATATC**CTA**CTTATCGTCGTCATCCTTGTAATCGATATC-3′) that encoded the FLAG tag and a STOP codon (in bold) and were flanked by EcoRV restriction sites (underlined). These were annealed, the product EcoRV digested and inserted into ‘pcDNA5-CTerm’ that had also been EcoRV digested. This generated the final pcDNA5-CTermFLAG, which contained the initiator Met codon, the final 67 terminal amino acids (from amino acids 837 to 903) of the human mitochondrial LARS2 followed by the 8 amino acids of the FLAG tag. All constructs were confirmed by restriction digest mapping and sequence analysis. Products were linearised to allow integration and stable transfection. Co-transfections were performed using Superfect (Qiagen, Manchester, UK) following manufacturer's recommendations. In all cases both vectors were linearized and combined in a 1:9 ratio (pcDNA6/TR:pcDNA5/FRT/TO/.

**The paper explained**

**Problem**

Mitochondrial DNA (mtDNA) disease has been recognized for over 20 years. As diagnostic tools have been refined, it is clear that mtDNA disease should no longer be regarded as a rare disorder. The majority of pathogenic mutations occur in the mtDNA-encoded mt-tRNAs, with more than 200 independent mutations having been described. These result in defects of mitochondrial protein synthesis, causing aberrant oxidative phosphorylation and a panoply of clinical presentations. To date, there is no effective cure for these disorders.

**Results**

Each aminoacyl tRNA synthetase has a specificity for its cognate tRNA. This process of recognition ensures that tRNAs are charged with the correct amino acid and that the fidelity of DNA sequence to protein composition is maintained. In contrast to this we show that overexpression of a non-cognate mitochondrial aminoacyl tRNA synthetase can overcome the respiratory defect caused by an mt-tRNA mutation. The pathogenic mt-tRNA^val^ mutation, which causes a loss of transcript stability when it is not charged with the correct amino acid, can be suppressed by the full length leucyl tRNA synthetase (LARS2) but not by alanyl, or phenylalanyl tRNA synthetases. This more generalized affinity of LARS2 for non-cognate mt-tRNAs is weaker than its affinity for its cognate mt-tRNA^leuUUR^ and mt-tRNA^leuCUN^ but is sufficient to be detected by cross linking and immunoprecipitation. Moreover, we have identified that this same mt-tRNA binding pattern is achieved with a short peptide corresponding to the C-terminus of LARS2.

**Impact**

Gene therapy with individual mitochondrial aminoacyl tRNA synthetases is theoretically a possibility but not an excessively practical one. Having a single therapeutic agent that could ameliorate mtDNA disease caused by any mt-tRNA mutation would be a huge step forward in terms of treatment. The possibility of using a short peptide that can intrinsically target mitochondria and effect therapeutic potential on all 22 mt-tRNA substrates is worthy of further investigation.

### Identification of oligoribonucleotides bound *in vivo*

To identify physiological interactions between proteins and mitochondrial RNAs cross linking immunoprecipitation (CLIP) was carried out essentially as previously described (Dennerlein *et al*, 2010). To generate a more comprehensive data set, the final PCR products were prepared for Ion Torrent sequencing, following manufacturer's instructions, rather than subcloning into pCR4-TOPO. Sequence data for 100 000–190 000 reads was collected, aligned to mtDNA as a reference sequence using the Torrent Suite software on the Ion Torrent server. The alignments were then viewed using IGV (integrative genomics viewer) and presented against a circular depiction of human mtDNA, using a log scale to indicate the number of reads per RNA site.

## For more information

http://www.mitoresearch.org.uk

http://www.mitochondrialncg.nhs.uk/

http://www.ncl.ac.uk/giving/what/health/mitochondria/
