# Supplementary material for: Human mitochondrial leucyl tRNA synthetase can suppress non cognate pathogenic mt-tRNA mutations
Source: EMBO Mol Med. 2014 Jan 10;6(2):183–93. doi: 10.1002/emmm.201303202 (PMC3927954; doi:10.1002/emmm.201303202)
Supplement: Supplementary file 1 [file emmm0006-0183-sd1.pdf]

EMBO Molecular Medicine

Human mitochondrial leucyl tRNA synthetase can suppress non cognate pathogenic mt-tRNA mutations

Hue Tran Hornig-Do, Arianna Montanari, Agata Rozanska, Helen A. Tuppen, Abdulraheem A. Almalki, Dyg P. Abg-Kamaludin, Laura Frontali, Silvia Francisci, Robert N. Lightowlers and Zofia M. Chrzanowska-Lightowlers

Corresponding authors: *Zofia M. Chrzanowska-Lightowlers and Robert N. Lightowlers, Newcastle University*

Review timeline:

Submission date:	19 June 2013
Editorial Decision:	22 July 2013
Additional author correspondence:	07 August 2013
Additional Editorial Correspondence:	07 August 2013
Additional author correspondence:	07 August 2013
Revision received:	23 August 2013
Editorial Decision:	16 October 2013
Revision received:	02 November 2013
Editorial Decision:	04 November 2013
Accepted:	07 November 2013

Transaction Report:

(Note: With the exception of the correction of typographical or spelling errors that could be a source of ambiguity, letters and reports are not edited. The original formatting of letters and referee reports may not be reflected in this compilation.)

Editor: Roberto Buccione

1st Editorial Decision 22 July 2013

Thank you for the submission of your manuscript to EMBO Molecular Medicine.

We have now received comments from the two out of the three Reviewers whom we asked to evaluate your manuscript. We are however experiencing some delay in obtaining the third.

Hence, to avoid further delays, I am sending the two consistent evaluations of Reviewers 1 and 2 at this time. I will forward Reviewer 3's delayed report, as soon as we are able to obtain it. When this report does arrive and if it raises additional important issues that have to be addressed to support this study, these would also need to be taken into consideration in your revision. I would not ask you to consider substantially further-reaching requests with respect to the current evaluations.

You will see that both Reviewers are generally supportive of your work and underline its considerable potential interest. Reviewer 1 raises a few points that require your intervention and thus prevent us from considering publication at this time. I will not dwell into much detail, as the comments are self-explanatory.

While publication of the paper cannot be considered at this stage, we would be pleased to consider a suitably revised submission, provided, however, that the Reviewers' concerns are fully addressed

with further experimentation where required.

Please note that it is EMBO Molecular Medicine policy to allow a single round of revision only and that, therefore, acceptance or rejection of the manuscript will depend on the completeness of your responses included in the next, final version of the manuscript.

I look forward to receiving your revised manuscript as soon as possible.

***** Reviewer's comments *****

Referee #1 (Remarks):

It has been shown by others that overexpression of mitochondrial tRNA synthetases (RS's) can ameliorate function in cells containing a mutation in both the cognate, and noncognate, mitochondrial tRNA. In this manuscript, the authors begin to address the mechanism by which such rescue might occur. First, they confirm these results with leucyl RS, but more importantly, they show that only the C-terminus (~70 aa) of LARS2 can rescue both the cognate and at least one non-cognate tRNA, but that the C-termini of at least two other RS's (Ala and Phe) cannot. This is an important - and in the case of the non-cognate rescue, surprising - finding that has both basic and translational ramifications. The work is extremely well done, and the discussion is both illuminating and thought-provoking.

In the methods, I did not delve into the constructs in detail, so I may have missed it, but you need to explain better (or show with a figure) what the 67-aa (I assume this is aa 837-903?) construct looks like. Since aa 837-903 of human LARS2 starts with a Glu (i.e. 837-EVV...LVQD-903), where is the initiator Met? If from the vector, are there any other amino acids prior to aa-835 that could have targeted the C-term to mitos? Please clarify this.

In the same vein, I presume that the authors believe that this C-terminal peptide goes to the inner membrane or matrix. Please show where it goes by submitochondrial fractionation, either "cold" (e.g. with appropriate antibodies, or with a C-terminal-epitope tag) or "hot" (e.g. with 35S-Met-labeled C-term).

The CLIP data imply that the C-terminus can interact with both the AARS2 and FARS2 (~50 RNA reads), which is not that much lower than the VARS2 level (~100 reads). How do these data reconcile with the observation of no rescue with AARS2 or FARS2?

Following up on a comment on p. 7 in the Discussion about conservation of RS's, it might be helpful to show the conservation of primary structure (by aa alignment) and/or secondary structure (by hydropathy alignment) among the various relevant RS's described in the paper.

Referee #2 (Remarks):

This study shows that human mitochondrial leucyl-tRNA synthetase and its C-terminal peptide can rescue the defects of non-cognate mutant tRNA. The molecular mechanism of the rescue is clearly demonstrated by showing that the overexpression stabilizes the mutant tRNA, leading to increases in OXPHOS complexes. Furthermore, it is shown that not all mitochondrial synthetases have the same rescue abilities. Also, the discussion of the properties of the C-terminal peptide is interesting and clarifying. Overall, this data is important for the potential use of synthetase derivatives as therapy for mitochondrial tRNA disorders.

Minor comment:
Figure 3B: AARS2 not AARS.

Additional author correspondence 07 August 2013

Thank you for your email of 22nd July 2013.

Our main reason for contacting you at this juncture is that we wondered whether the third reviewer had responded and whether there any other comments we need to consider before re-submitting our manuscript. Your previous email indicated "I would not ask you to consider substantially further-reaching requests with respect to the current evaluations." We were not sure if this meant that no major points over and above those from reviewers 1 and 2 would need attention. If we complete our amendments will we still need to wait for reviewer 3 before we resubmit our manuscript ? Perhaps you could guide us on this point ?

In response to the reviewers' comments, we have been amending our manuscript. All the points have been addressed apart from the comment on the submitochondrial localisation. The original manuscript presents CLIP data that demonstrates physiological binding of the LARS2 C-terminus to mt-tRNAs that are only present in the mitochondrial matrix. Further, IP studies show that we were able to detect, albeit at low level, the peptide in protease-shaved mitochondria. The reviewers do not appear to doubt the CLIP data or the presence of the peptide in mitochondria but reviewer 1 suggests that further sublocalisation would enhance the manuscript. We are working towards showing this, but there are recognised technical difficulties with detecting such small peptides in samples from which 5 separate fractions need to be obtained.

Finally, we have been in contact with the authors of Perli et al and understand that the experiments that they plan may still take a little while. However, if we are both able to successfully address the comments from the reviewers of both manuscripts, we would still aim for the papers to be published together.

Additional Editorial Correspondence 07 August 2013

Thank you for your letter.

What I meant in my decision letter was that only significant caveats, if raised by the missing Reviewer would have needed to be addressed.

That said, however, I can formally confirm that we are not expecting nor will we be considering the third evaluation at this point. Therefore please do continue with your revision based on the two available evaluations

As for the issue of submitochondrial localisation, the point you make is well-taken and I agree that the Reviewers did not appear to doubt the presence of the peptide in mitochondria. Although I cannot commit on the Reviewers' behalf, I would suggest that you develop your study in this sense only as far as realistically possible.

Finally, I do agree that ideally, it would be much preferable if you proceeded hand in hand with Prof. d'Amati's group so that the papers could be eventually published together.

Additional author correspondence 07 August 2013

We could not have asked for a faster or fairer response ! Thank you.

We will still try to generate more data on the submitochondrial localisation and then we will submit a revised manuscript. The first author is returning from Germany to Newcastle to perform these experiments but we would anticipate being able to resubmit before the end of the month.

1st Revision - authors' response 23 August 2013

We thank the reviewers for their time and useful comments and apologise for areas where our manuscript has left our meaning or results unclear. We hope that we have rectified this and that the manuscript is now acceptable for publication. We have outlined our responses to the reviewers below and submitted a 'tracked changes' version as well as a final version, to make it easier for the reviewers to register the alterations that we have made.

Reviewer 1.

Comment

"*In the methods, I did not delve into the constructs in detail, so I may have missed it, but you need to explain better (or show with a figure) what the 67-aa (I assume this is aa 837-903?) construct looks like. Since aa 837-903 of human LARS2 starts with a Glu (i.e. 837-EVV...LVQD-903), where is the initiator Met? If from the vector, are there any other amino acids prior to aa-835 that could have targeted the C-term to mitos? Please clarify this.*"

Response

We apologise for the lack of clarity in the description of these constructs. We have now increased the detail to indicate how the construct was generated with an initiator methionine preceding the final 67 amino acids of LARS2 and a FLAG tag following.

There is only the addition of a single methionine prior to the 67 amino acids of the C-terminus with no other extraneous sequence that could influence targeting to mitochondria.

Comment

"In the same vein, I presume that the authors believe that this C-terminal peptide goes to the inner membrane or matrix. Please show where it goes by submitochondrial fractionation, either "cold" (e.g. with appropriate antibodies, or with a C-terminal-epitope tag) or "hot" (e.g. with 35S-Met-labeled C-term)."

Response

We had originally included the immunoprecipitations from mitochondria together with the CLIP data that showed interactions with mitochondrial tRNAs as evidence of intramitochondrial localisation. In response to the reviewer's comments we have, as suggested, now performed the "cold" experiment. We have used western analysis on proteinase K shaved mitoplasts, to show more clearly that the C-terminal fragment is with the matrix/imm compartment. We have included a new figure and amended the text to indicate this.

Comment

"The CLIP data imply that the C-terminus can interact with both the AARS2 and FARS2 (~50 RNA reads), which is not that much lower than the VARS2 level (~100 reads). How do these data reconcile with the observation of no rescue with AARS2 or FARS2?"

Response

I fear that there may have been some confusion on this point. The CLIP data reflects the interaction of the full length or C-terminus of LARS2 with the mitochondrial RNA species. It does not reflect interactions between the C-terminus of LARS2 and either AARS2 or FARS2 protein, as the reviewer's comment seems to suggest. Perhaps the reviewer meant that the CLIP data shows an interaction with the mt-tRNAAla and Phe ?

The only mutant and unstable mt-tRNA in the cell line is the mt-tRNA valine. The C-

terminus and indeed the full length LARS2 can interact with a number of RNA species as seen by the CLIP data indicating the regions of mtRNA that interact with the LARS2 protein but the interaction will only have a positive and beneficial effect on the stability of mt-tRNA valine, hence the improvement in the cell viability and respiratory capacity.

Binding of the LARS2 to all the other mt-tRNAs that are wild type has no detrimental effect. The expression of AARS2 or FARS2 was elicited in the cell line that harboured the mt-tRNA valine mutation and were intended to be used as a negative controls to show that respiratory rescue could not simply be effected by the overexpression of any mitochondrial tRNA synthetase. Overexpression of neither AARS2 nor FARS2 proteins demonstrated any therapeutic binding to the mt-tRNA valine, as the cells remained respiratory compromised.

I have made a few amendments to the text and hope that this now makes clearer the reason for the lack of rescue observed during AARS2 and FARS2 expression.

Comment

Following up on a comment on p. 7 in the Discussion about conservation of RS's, it might be helpful to show the conservation of primary structure (by aa alignment) and/or secondary structure (by hydropathy alignment) among the various relevant RS's described in the paper.

Response

In this response to the reviewer's comment we have analysed the final 67 amino acids of the 4 aaRSs used in this study by looking at the hydropathy plots and hydropathy clusters. PDB entries for the human aaRS proteins are only available for the FARS2, so we have used a simple web based tool (GOR4) that determines structural features predicted from the amino acid sequences. These composite data are attached to this response but we do not feel that the manuscript would benefit from their inclusion.

Overall when looking for trends in the hydropathy plots the LARS2 and the VARS2 proteins appear more similar to each other than to either AARS2 or FARS2. This pattern is also true when looking at the trends of the structural predictions. The hydropathy clusters show little obvious trend.

We have added a sentence to the discussion indicating that no immediate striking similarities could be seen between LARS2 and VARS2 but that more detailed analysis of tertiary structures by comparing known structures for similar proteins will form part of the future investigations, particularly by our Italian colleagues.

Reviewer 2.

Comment

Minor comment:

Figure 3B: AARS2 not AARS.

Response

This has now been amended.

Hydropathy plots (left), clusters (upper right) and structural predictions (lower right) of C-termini of mt-aaRS2s

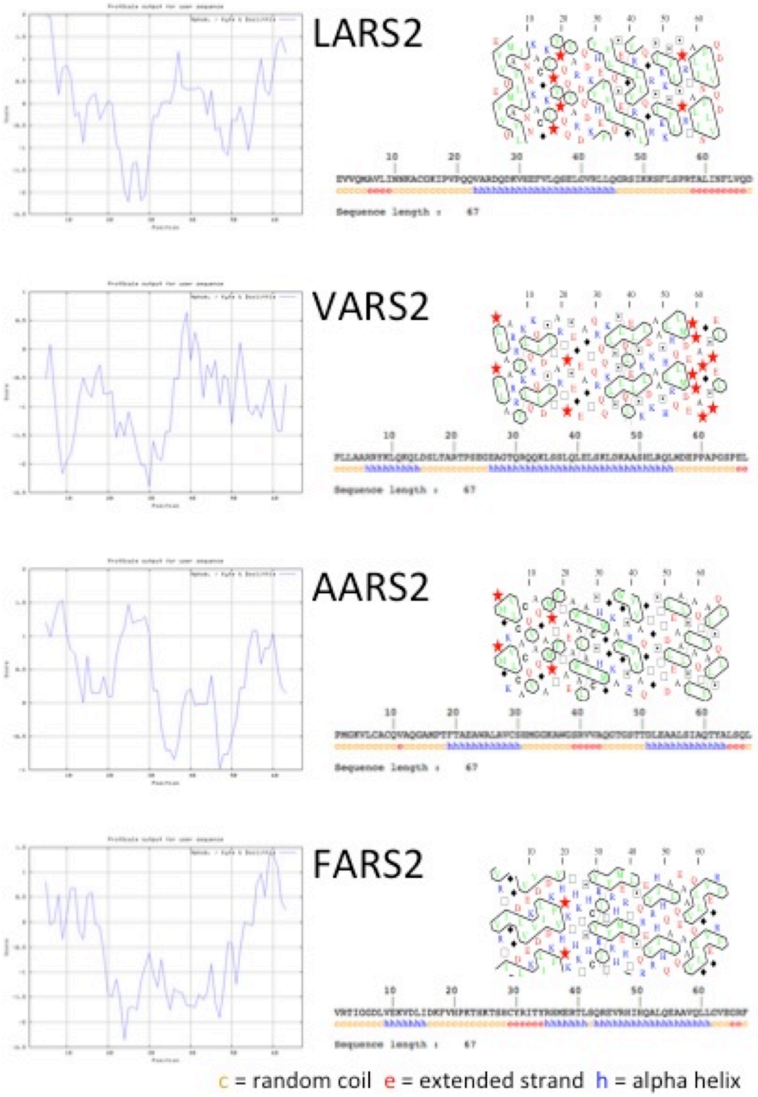


```
Q15031|LARS2_HUMAN  EVVQMAVLINNKACGKIPVPQQVARDQDKVHEFVLQSELGVRLLQGRSIKKSFLSP
Q5ST30|VALRS2_HUMAN -PLLAARRYKLQKQLDSLTARTPSEGEAGTQRQQKLSSLQLELSKLDKAASHLRQL
Q5JTZ9|ALARS2_HUMAN -PMGKVLCACQVAQGAMPTFTAEAWALAVCSHMGGKAWGSRVVAQGTGSTTDLEAAI
O95363|FARS2_HUMAN  VRTIGGDLVEKVDLIDKFVHPKTHKTSHCYRITYRHMERTLSQREVRHIHQALQEA
                                           .                               :         :
```

Hydrophobicity prediction of transmembranous or globular proteins.

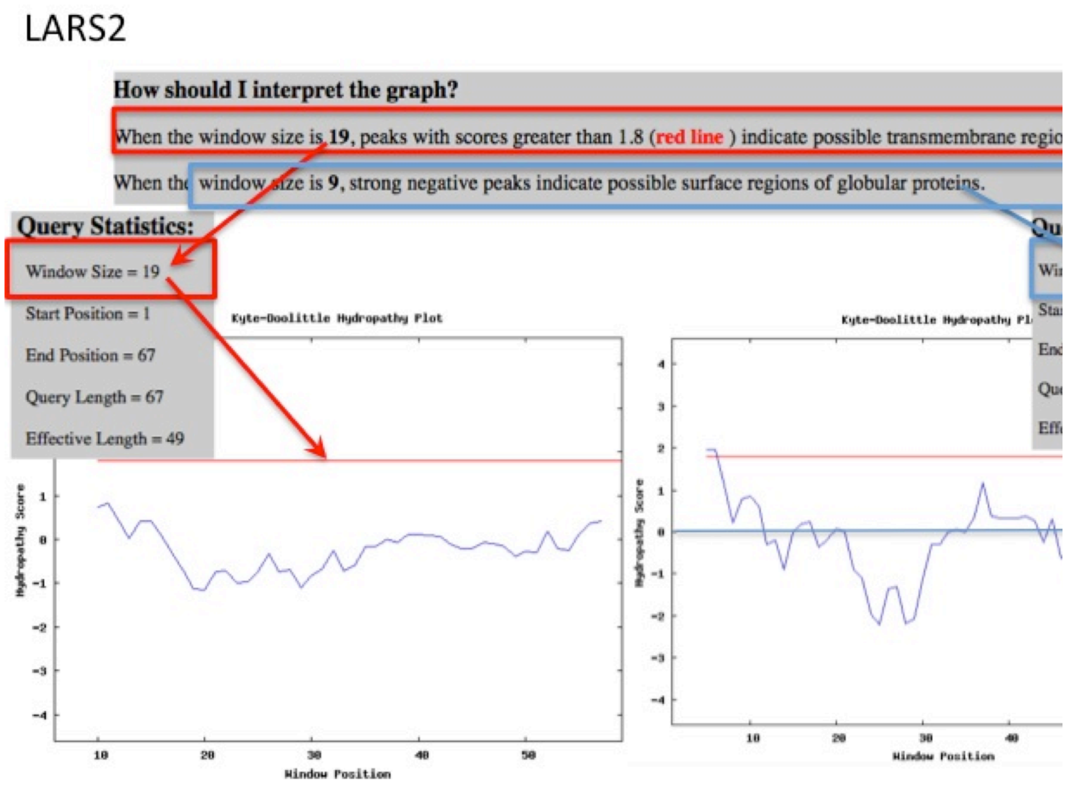

http://gcat.davidson.edu/DGPB/kd/kyte-doolittle.htm

2nd Editorial Decision 16 October 2013

Thank you for the submission of your revised manuscript to EMBO Molecular Medicine. The Reviewer who was asked to re-assess it is now supportive and I am pleased to inform you that we will be able to accept your manuscript pending the following final amendments:

As per our Author Guidelines, the description of all reported data that includes statistical testing must state the name of the statistical test used to generate error bars and P values, the number (n) of independent experiments underlying each data point (not replicate measures of one sample), and the actual P value for each test (not merely 'significant' or 'P < 0.05').

Please submit your revised manuscript within two weeks. Obviously, the sooner you send us the revised version, the sooner we will be able to accept it for publication.

I look forward to seeing the next, final version.

***** Reviewer's comments *****

Referee #1 (Remarks):

No comments

2nd Revision - authors' response 02 November 2013

Thank you for your recent email indicating that our paper would now be accepted upon addition of the p values where statistical significance was documented. This information has now been added and the final version of the manuscript submitted with this letter.

We also thank the reviewers for re-examining our manuscript.

3rd Editorial Decision 04 November 2013

Thank you for providing a set of figure files with improved resolution.

The figure files are now satisfactory and we are now ready to accept your manuscript but for one small remaining issue. Although we had asked you to provide actual P values for each test (not merely 'significant' or 'P < 0.05'), we notice that (see figure legends) this has not been done. Please check carefully and amend as necessary.

As we will be implementing our new online platform soon (January 2014), we will now be needing a short list (up to 5) of bullet points that summarize the key NEW findings to accompany papers. The bullet points should be designed to be complementary to the abstract and will be used online.

Finally, I thought you might like to know that I have asked a Leader in the field to write a Closeup article on your and Prof. D'Amati's work. Closeups are short commentaries (our version of News and Views) designed to emphasize the novelty and broader implications of selected articles. We will be including the Closeup piece and your articles in the same issue (to be determined).

Please submit your final revised manuscript within two weeks. Needless to say, the earlier the better.

I look forward to reading a new revised version of your manuscript as soon as possible.
