# Supplementary material for: Human mitochondrial leucyl tRNA synthetase can suppress non cognate pathogenic mt-tRNA mutations
Source: EMBO Mol Med. 2014 Jan 10;6(2):183–93. doi: 10.1002/emmm.201303202 (PMC3927954; doi:10.1002/emmm.201303202)
Supplement: Supplementary file 2 [file emmm0006-0183-sd2.pdf]

Supplementary Information

Figure S1

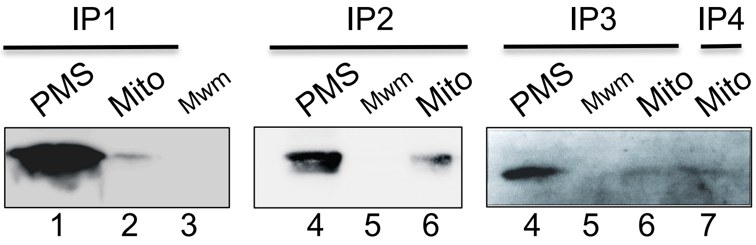

Figure S1
The C-terminus of LARS2 can be immunoprecipitated from mitochondria via the FLAG tag.
Cells were induced to overexpress (3 days) a FLAG tagged C-terminal fragment of LARS2 (n = 4). Mitochondria (Mito) were isolated and the post mitochondrial supernatant (PMS) retained. Anti-FLAG mediated immunoprecipitations (IP1, 2, 3 and 4) were performed on each fraction and the eluates separated by 16% Tricine-SDS PAGE. Immunoblotting with antibodies to the FLAG moiety revealed a small but consistent proportion of C-terminal fragment within the mitochondria. Molecular weight markers (Mwm) were loaded alongside but are not reactive to the secondary or ECL+ reagents.
